# Identification of non-recurrent right inferior laryngeal nerve during thyroidectomy

**DOI:** 10.1093/jscr/rjaf971

**Published:** 2025-12-05

**Authors:** Gerardo D’Amato, Mario Musella, Carolina Bartolini, Lucrezia Borrelli, Alessandra D’Ambrosio, Antonio Franzese, Vincenzo Schiavone, Pasquale Avella, Mafalda Ingenito

**Affiliations:** Università degli Studi di Napoli “Federico II”, Dipartimento di Scienze Biomediche Avanzate, Campania, Napoli 80131, Italy; Università degli Studi di Napoli “Federico II”, Dipartimento di Scienze Biomediche Avanzate, Campania, Napoli 80131, Italy; Università degli Studi di Napoli “Federico II”, Dipartimento di Scienze Biomediche Avanzate, Campania, Napoli 80131, Italy; Università degli Studi di Napoli “Federico II”, Dipartimento di Scienze Biomediche Avanzate, Campania, Napoli 80131, Italy; Università degli Studi di Napoli “Federico II”, Dipartimento di Scienze Biomediche Avanzate, Campania, Napoli 80131, Italy; Università degli Studi di Napoli “Federico II”, Dipartimento di Scienze Biomediche Avanzate, Campania, Napoli 80131, Italy; Università degli Studi di Napoli “Federico II”, Dipartimento di Scienze Biomediche Avanzate, Campania, Napoli 80131, Italy; Università degli Studi di Napoli “Federico II”, Dipartimento di Scienze Biomediche Avanzate, Campania, Napoli 80131, Italy; Università degli Studi di Napoli “Federico II”, Dipartimento di Scienze Biomediche Avanzate, Campania, Napoli 80131, Italy

**Keywords:** non-recurrent inferior laryngeal nerve, thyroidectomy, toxic thyroid goiter

## Abstract

The non-recurrent right inferior laryngeal nerve is a rare anatomical variant of the recurrent laryngeal nerve, almost always associated with an aberrant right subclavian artery (Arteria Lusoria). Its unusual course increases the risk of iatrogenic injury during thyroid and neck surgery. A 45-year-old woman underwent right hemithyroidectomy for a solitary thyroid nodule suggestive of a follicular tumor. The recurrent laryngeal nerve was not found in its expected tracheoesophageal location. Careful dissection of the vagus nerve revealed a non-recurrent branch arising near the thyroid superior pole and coursing horizontally to the larynx. The nerve was preserved, and surgery was completed uneventfully without intraoperative neuromonitoring. The non-recurrent right inferior laryngeal nerve lies higher and more anterior than the recurrent pathway strongly linked to Arteria Lusoria. Failure to recognize it may cause permanent vocal cord paralysis. Preoperative imaging may suggest its presence, but intraoperative identification through meticulous vagus dissection remains essential for safe surgery.

## Introduction

The inferior laryngeal nerve (ILN), more commonly known as the recurrent laryngeal nerve (RLN), is a branch of the vagus nerve (X cranial nerve) that provides motor and sensory innervation to the intrinsic muscles of the larynx, except for the cricothyroid muscle. Generally, on the right side, it loops around the subclavian artery before ascending in the tracheoesophageal groove, while on the left side, it loops around the arch of the aorta [1]. In the non-recurrent variant, instead, the right ILN arises directly from the vagus nerve in the neck and courses horizontally toward the larynx, without descending into the chest [2]. This anomaly is almost always linked to an aberrant right subclavian artery (called Arteria Lusoria). Though rare, its recognition is critical, as inadvertent injury can cause significant morbidity. The prevalence of NRR-ILN is estimated at around 0.5% in thyroid surgery patients. It is almost always found on the right side due to the embryological development of the great vessels and the associated aberrant right subclavian artery. Left-side are extremely rare and typically associated with situs inversus totalis [3].

## Case presentation

A 45-year-old woman came to our attention for a symptomatic solitary nodule in the right thyroid lobe. Fine-needle aspiration suggested a follicular tumor, and preoperative ultrasound confirmed a solid 3 cm lesion. She had no prior history of neck surgery, nor was radiation reported.

A right hemithyroidectomy was performed. During a careful dissection and mobilization of the right thyroid lobe, the expected recurrent course of the ILN in the tracheoesophageal groove could not be identified. So, the cricothyroid joint was exposed, but no nerve was found in its usual recurrent course ascending from the thoracic inlet. Suspecting an anatomical variation, the surgical team carefully explored the field, tracing the vagus nerve within the carotid sheath. A nerve branch arising directly from the vagus nerve at the level of the thyroid’s superior pole was identified, coursing horizontally and cranially into the larynx, confirming the presence of a non-recurrent right inferior laryngeal nerve (NRR-ILN). The nerve was carefully preserved, and the procedure was completed without complications. Intra-operative neuromonitoring was not used; instead, anatomical dissection was the primary technique for identification.

## Discussion

The identification of an NRR-ILN is a critical challenge for surgeons. Due to its unusual course, it is often not found in the traditional location in the tracheoesophageal groove, making it highly susceptible to iatrogenic injury.

**Figure 1:**
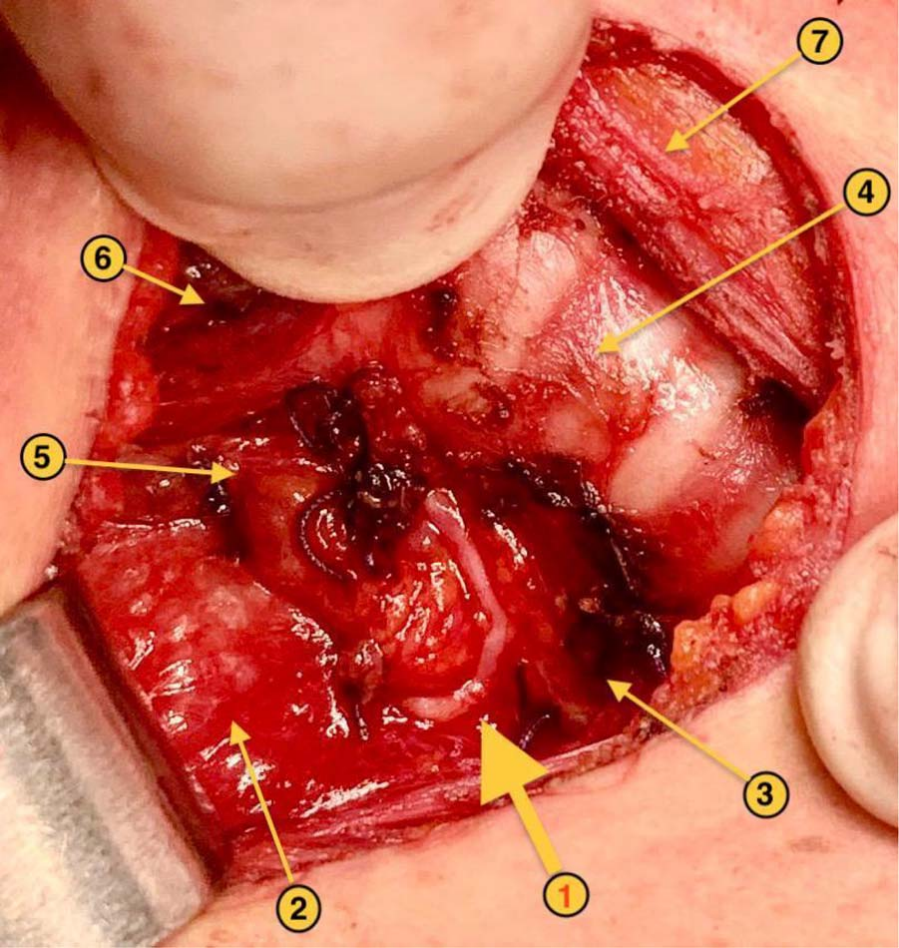
NRR-ILN: anatomical landmarks. 1. Right ILN. 2. Right cervical neurovascular bundle. 3. Esophagus. 4. Trachea. 5. Right superior parathyroid gland. 6. Right cricothyroid muscle. 7. Left sternothyroid muscle.

Generally, the right ILN originates as a branch of the vagus nerve in the thorax, near the pleural apex. From there, it loops around the right subclavian artery, then ascends in the tracheoesophageal groove to reach the larynx. This “backward” course (hence the name recurrent) is the norm. In the case of the NRR-ILN, the nerve arises directly from the vagus nerve in the neck and proceeds in a more or less horizontal or oblique course, heading antero-superiorly toward the cricothyroid muscle. Its position is therefore higher and more anterior than the classical course, making it more vulnerable during the initial phases of surgical dissection, especially during the mobilization of the superior and middle thyroid lobes (Fig. 1) [4, 5].

The occurrence of an NRR-ILN is strongly associated with Arteria Lusoria, an aberrant right subclavian artery. During embryonic development, the ILN courses beneath the right subclavian artery. Typically, the left fourth branchial artery (which will give rise to the proximal part of the aortic arch) and the right fourth branchial artery (which will give rise to the proximal part of the right subclavian artery) form two rings. The sixth branchial artery contributes to the ductus arteriosus and the initial part of the pulmonary artery. Due to an anomaly in the development of the aortic arch and the subclavian arteries, in rare cases, the right subclavian artery doesn’t originate from the right fourth branchial arch but anomalously arises from the distal aorta, taking a retroesophageal and retrotracheal course. This aberrant pathway is known as Arteria Lusoria. Because the right subclavian artery develops in an abnormally distal position, the right ILN lacks a vascular loop around which to recur. Consequently, the nerve follows a direct, non-recurrent course, arising from the vagus nerve in the neck and proceeding straight to the larynx [6, 7]. Recognition of one anomaly should immediately raise suspection for the other.

A lack of awareness of this variant and its link to the Arteria Lusoria can lead the surgeon to search for the ILN in its classical path, in the tracheoesophageal groove. Not finding it, they might consider the nerve absent or retracted, proceeding with the dissection and risking an inevitable injury. Maintaining a high index of suspicion is essential to prevent injury to NRR-ILN. When the nerve is not found in its expected recurrent position, the surgeon must immediately consider this anatomical variant. The dissection should be halted, and the vagus nerve should be meticulously identified and traced caudally from the superior pole of the thyroid. The NRR-ILN will typically be found branching directly from the vagus nerve and running horizontally or antero-superiorly to the larynx. The use of intraoperative nerve monitoring (IONM) can be a valuable adjunct, but it should not replace careful anatomical dissection [8].

Failure to recognize and preserve the NRR-ILN can lead to serious complications. Unilateral injury results in vocal cord paralysis, causing hoarseness, dysphonia, and an increased risk of aspiration. Bilateral injury, while rare, can result in complete laryngeal paralysis, leading to airway obstruction requiring an emergency tracheostomy. The morbidity associated with NRR-ILN injury is directly related to the high probability of permanent vocal cord paralysis, as the nerve may be transected rather than stretched, reducing the chance of recovery [9, 10].

## Conclusion

The NRR-ILN is a rare but clinically significant anatomical variation that every surgeon performing neck surgery should be aware of. Its presence should be suspected when the expected ILN is not identified in the tracheoesophageal groove. Preoperative imaging, although not always conclusive, may sometimes suggest the presence of an aberrant right subclavian artery. The key to preventing injury is meticulous anatomical dissection, a high index of suspicion, and the willingness to deviate from the standard search pattern when the nerve is not found in its usual location. Early recognition and careful preservation of this nerve are paramount to ensure optimal patient outcomes and prevent life-altering complications.
